# Combined Transcutaneous Electrical Spinal Cord Stimulation and Task-Specific Rehabilitation Improves Trunk and Sitting Functions in People with Chronic Tetraplegia

**DOI:** 10.3390/biomedicines11010034

**Published:** 2022-12-23

**Authors:** Niraj Singh Tharu, Monzurul Alam, Yan To Ling, Arnold YL Wong, Yong-Ping Zheng

**Affiliations:** 1 Department of Biomedical Engineering, The Hong Kong Polytechnic University, Hong Kong SAR, China; 2 Department of Rehabilitation Sciences, The Hong Kong Polytechnic University, Hong Kong SAR, China; 3 Research Institute for Smart Ageing, The Hong Kong Polytechnic University, Hong Kong SAR, China

**Keywords:** transcutaneous electrical spinal cord stimulation, trunk control, sitting balance, tetraplegia, spinal cord injury

## Abstract

The aim of this study was to examine the effects of transcutaneous electrical spinal cord stimulation (TSCS) and conventional task-specific rehabilitation (TSR) on trunk control and sitting stability in people with chronic tetraplegia secondary to a spinal cord injury (SCI). Five individuals with complete cervical (C4–C7) cord injury participated in 24-week therapy that combined TSCS and TSR in the first 12 weeks, followed by TSR alone for another 12 weeks. The TSCS was delivered simultaneously at T11 and L1 spinal levels, at a frequency ranging from 20–30 Hz with 0.1–1.0 ms. pulse width biphasically. Although the neurological prognosis did not manifest after either treatment, the results show that there were significant increases in forward reach distance (10.3 ± 4.5 cm), right lateral reach distance (3.7 ± 1.8 cm), and left lateral reach distance (3.0 ± 0.9 cm) after the combinational treatment (TSCS+TSR). The stimulation also significantly improved the participants’ trunk control and function in sitting. Additionally, the trunk range of motion and the electromyographic response of the trunk muscles were significantly elevated after TSCS+TSR. The TSCS+TSR intervention improved independent trunk control with significantly increased static and dynamic sitting balance, which were maintained throughout the TSR period and the follow-up period, indicating long-term sustainable recovery.

## 1. Introduction

Spinal cord injury (SCI) often results in a permanent loss of motor, sensory, or autonomic functions below the level of injury [1,2]. The worldwide annual incidence and prevalence of SCI range from 8 to 246 and from 236 to 1298 per million people, respectively [3]. Depending on the level of neurological damage, SCI may cause tetraplegia or paraplegia [4]. One-third of people with SCI are found to have tetraplegia, and 50% of them have a complete lesion [5], with C5 (cervical vertebrae) being the most commonly affected level [6]. People with tetraplegia experience severe trunk muscle weakness that inhibits sitting and trunk functions, thus restricting their overall functional activity [7]. In addition, individuals with complete or incomplete tetraplegia have greater trunk impairment, which results in more trunk instability and sitting imbalance than people with paraplegia [8,9], and often struggle to perform the daily functions of sitting [10,11]. They are also more prone to postural instability and fall-related injuries [12]. For people with tetraplegia, trunk stability is more important than trunk mobility [13]. Individuals with tetraplegia report significantly more limitations and restrictions in their activities of daily living (ADL) than people with paraplegia [14]. Similarly, more than 60% of SCI survivors consider trunk function to be an important factor for mobility and performing ADL [15]. Around 70–80% of people with SCI are wheelchair-bound, and consider trunk control an essential factor for those with sitting difficulties [16].

Traditionally, SCI management focused on teaching compensatory skills. Recently, this approach has gradually shifted toward neural restoration through the use of innovative, intensive therapy strategies that successfully address physiological changes and regeneration concepts [17,18]. Of various approaches (activity-based therapy, impairment-based training, etc.), task-specific rehabilitation (TSR) has been shown to be more beneficial than conventional rehabilitation for motor recovery in people with chronic SCI [19,20]. “The TSR is a therapeutic approach that involves intensive practice of actions or functional tasks” [21], which is based on the idea that motor output can be shaped and retrained in response to specific sensory inputs [22]. It focuses on regaining muscular strength and improving functional abilities through targeted and repeated specific exercises [23]. Motor or sensory recovery is often restricted to minimal success in people with tetraplegia [24,25]. In addition, TSR has been observed to be advantageous for improving upper limb functions [26]. The locomotion ability was reported to be enhanced in individuals with incomplete SCI using TSR strategies [27,28]. Although TSR has shown some benefits over conventional rehabilitation, more studies are needed to confirm its efficacy [29,30].

It used to be believed that people with complete SCI would not experience motor recovery below the lesion [31]. Yet prior research has indicated that individuals with complete or incomplete SCI showed motor and sensory improvement with neuromodulation treatment [32]. In addition, transcutaneous electrical spinal cord stimulation (TSCS) has recently emerged as a potential treatment for SCI, among other neuromodulation techniques [33,34]. It modulates the activity of neural pathways through spinal stimulation to generate therapeutic benefits [35]. The TSCS produces an electric field that triggers the neural connectome via sensory pathways in the dorsal roots, which provides subthreshold excitation to interneurons and motor neurons distal to the injury. Motor neurons near the threshold are then more rapidly triggered and produce volitional movement [36]. Previous studies indicated that TSCS may decrease spasticity [37], modify neuronal connections [38], assist in locomotion [39] and stepping [40], and initiate volitional movement [41]. Moreover, TSCS produced immediate trunk self-control in people with chronic SCI with a more steady and upright sitting position [12], and quickly restored the ability to sit up straight [42]. However, these studies on trunk control have only been conducted on people with paraplegia or incomplete cervical SCI [11,43]. More research is warranted to investigate trunk recovery in people with complete tetraplegia [42].

The current study aimed to investigate the effects of adding TSCS to TSR treatment on trunk control and sitting function in people with tetraplegia following complete cervical SCI. To achieve this aim, two specific objectives were set to investigate the (1) efficacy of TSCS for improving trunk control and sitting function with TSR in people with tetraplegia; and (2) effect of TSCS on motor and sensory functions of the study participants. We hypothesized that the combined treatment (TSCS+TSR) could improve trunk and sitting functions in people with complete chronic tetraplegia. To our knowledge, this is the first study to report the use of TSCS for trunk control in people with complete cervical SCIs; the outcomes may be applicable to people with various levels of neurological damage caused by SCI.

## 2. Methods

The study was approved by the Human Subjects Ethics Sub-Committee of The Hong Kong Polytechnic University (Reference no: HSEARS20190201002-01).

### 2.1. Study Participants

Five individuals with chronic SCI with impaired trunk and sitting function were recruited. Each participant had sustained a traumatic complete cervical SCI (Table 1). Participant consent was obtained prior to the start of the experiment.

As shown in Table 1, the mean age of the participants was 42.0 years (SD = 13.7 years, range = 26–57 years), and the mean time since injury was 9.3 years (SD = 7.4 years, range = 1.5–19 years). Each participant had a traumatic cervical SCI based on the American Spinal Injury Association Impairment Scale (AIS), and the injury level varied from C4–C7. The inclusion criteria were people with SCI aged between 18 and 60 years; a cervical SCI resulting in complete tetraplegia; injury duration of at least 1 year; an injury level between C4–C8; absence of independent trunk control; being unable to sit independently; stable respiratory functions; and no prior neuromodulation treatment. People were excluded if they had received injections (Botox or Dysport) within the previous 6 months, had spasticity (more than grade 1^+^ based on the Modified Ashworth Scale), contracture, pressure injuries, infections, or internal fixations at the site of the injury, transplants (cardiac pacemakers and defibrillators), or had comorbidities (e.g., asthma, hypertension).

### 2.2. Study Design

**Figure 1:**
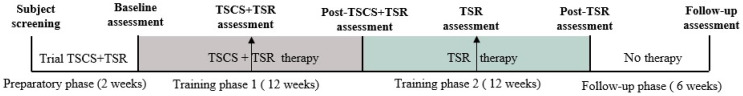
The outline of the study, divided into four phases: (i) The preparatory phase, in which participants were enrolled and screened to ensure their eligibility for the study. During this period, their response to TSCS was tested and optimal stimulation settings were determined. The visual analog scale was used to record their response to the stimulation. After completing a baseline assessment over a period of 2 weeks, the participants received two phases of therapy, each lasting for 12 weeks, with two half-way assessments completed every 6 weeks. (ii) Training phase 1, transcutaneous electrical spinal cord stimulation (TSCS) with task-specific rehabilitation (TSR); (iii) training phase 2, TSR alone; and (iv) the follow-up phase, during which no therapy was performed, and assessments were repeated 6 weeks following the completion of the TSR training period.

This was a case series study design where people with chronic tetraplegia underwent two phases of treatment (first TSCS+TSR followed by TSR alone). The participants were recruited through a convenience sampling method. The treatment frequency was divided into three groups depending on the availability and schedules of the study participants: (a) three sessions per week; (b) two sessions per week; and (c) one session per week. In addition, participants P1, P2, and P4 attended three sessions per week, whereas P3 and P5 attended one session and two sessions per week, respectively. The outline of the study is described in Figure 1.

### 2.3. Experimental Protocol

The 12th free rib was palpated and followed to identify the T11 and T12 vertebrae. The iliac crest was palpated and followed to the posterior superior iliac spine, and then to the L1 and L2 levels. Two stimulation electrodes were then attached between T11–T12 and L1–L2 spinous processes in the middle, targeting the spinal cord (hereinafter called T11 and L1 electrodes), while another two referencing electrodes were placed above the iliac crests bilaterally. For the active electrodes, we utilized a pair of self-adhesive electrodes with a size of 3.2 cm (ValuTrode, Axelgaard Manufacturing Co., Ltd., Fallbrook, CA, USA) and another pair of internally linked 6.0 × 9.0 cm self-adhesive rectangle-shaped electrodes (Guangzhou Jetta Electronic Medical Device Manufacturing Co., Ltd., Guangdong, China) as ground electrodes. The T11 and L1 regions of the study participants were stimulated using two specifically designed constant current stimulators (DS8R, Digitimer, UK). In order to activate the stimulators, a function generator (AFG1022, manufactured by Tektronix, Inc., Beaverton, OR, USA) was used to produce a burst of 10 kHz, which was transmitted at a frequency of 20–30 Hz [41]. The burst configuration was raised to 10 biphasic pulses (1.06 ms burst duration, henceforth referred to as 1 ms), and the pulse lengths of each cycle were maintained constant at 50 µs for both devices. The previous study of [41] showed that a shorter pulse of 0.5 ms was more effective in improving functional movements. In addition, it has been reported that short pulse durations (0.05–0.4 ms) preferentially activate motor axons, while the use of longer pulse durations (0.5–1 ms) activates sensory axons [43]. Therefore, the short pulse duration of 0.05 ms was chosen in the current study.

To determine the optimal stimulation intensity, the investigators used the Visual Analog Scale to measure the pain intensity. The participants were asked to experience involuntary extension of the trunk (straightening of the spine), and asked to feel when the trunk became more stable in the presence of the stimulation. Based on the individual participant’s response, he/she experienced the best trunk stability and tolerable stimulation intensity, which ranged between 95–115 milliamperes (mA), depending on each individual’s response. The other stimulation settings (such as frequency, carrier frequency, and burst configuration) were kept constant for all the participants throughout the experiment. The stimulation intensity varied between individual participants, which was set based on their response as described above. During every treatment session, the participant was asked to experience trunk extension with increased trunk stability in sitting as the intensity was gradually increased. A previous study demonstrated that stimulation of the rostral portion of the lumbar enlargement (corresponding to the T11–T12 vertebral level) at a frequency of 30 Hz could specifically facilitate voluntary movements, whereas stimulation delivered over the caudal area of the lumbar enlargement (corresponding to the L1–L2 vertebral level) at a frequency of 15 Hz would facilitate tonic extensor activity specific for postural control [12]. The 10 kHz carrier frequency was found to be suitable for stimulating the spinal circuits of both injured and uninjured individuals [44]. Therefore, the above-mentioned parameters were adopted for the stimulation settings. The TSR included spinal mobility exercises such as flexion, extension, lateral flexion, and rotation, as well as static and dynamic seated balancing exercises. The participants were trained in a variety of experimental settings and positions, ranging from sitting in a wheelchair to lying on a bed to lying on a floor mat, with the help and supervision of a physiotherapist.

For combinational treatment, after the placement of stimulating electrodes, the stimulation parameters were set and the intensity gradually increased until the individual’s maximum tolerance was reached. Then the participant was asked to perform various task-specific exercises. Each session lasted for 45–60 min, divided into three sub-sessions of 15–20 min each. A break was given after each sub-session, and the stimulation was turned off during the rest period. The participants were stimulated throughout each session, except during the break. Because all participants in the present study had complete tetraplegia, TSCS+TSR was delivered in the first 12 weeks to determine whether the combinational treatment could improve trunk and sitting functions. Another 12 weeks of TSR alone was followed to determine whether the functional gains could be maintained.

### 2.4. Outcome Measures

The functional assessment was conducted using the following outcome measures: (1) Modified Functional Reach Test (mFRT) to determine the functional reach distance; (2) Trunk Control Test (TCT) to assess static and dynamic balance (3) Function in Sitting Test (FIST) to measure functional sitting balance; and (4) International Standards for Neurological Classification of Spinal Cord Injury (ISNCSCI) to assess sensory and motor levels on both the right and left sides, the neurological degree of damage, and the completeness of the injury. The mFRT [45], TCT [46], and FIST [47] have proven reliability and validity for assessing people with SCI. The TCT comprises 0–24 points and FIST 0–56 points, where a higher score indicates improvement in function. Additionally, the trunk kinematics (i.e., range of motion, ROM) were assessed using a motion capture system (Vicon Nexus 2.5.1, Vicon Nexus TM, Vicon Motion Systems Ltd., Yarnton, UK) (ROM), while surface electromyography (EMG) (Model DE-2.1; Delsys USA, Inc., Boston, MA, USA) was used to the measure muscle activity of four pairs of trunk muscles (rectus abdominis, external oblique, erector spinae, and latissimus dorsi).

### 2.5. Vicon Marker and EMG Electrode Placement

**Figure 2:**
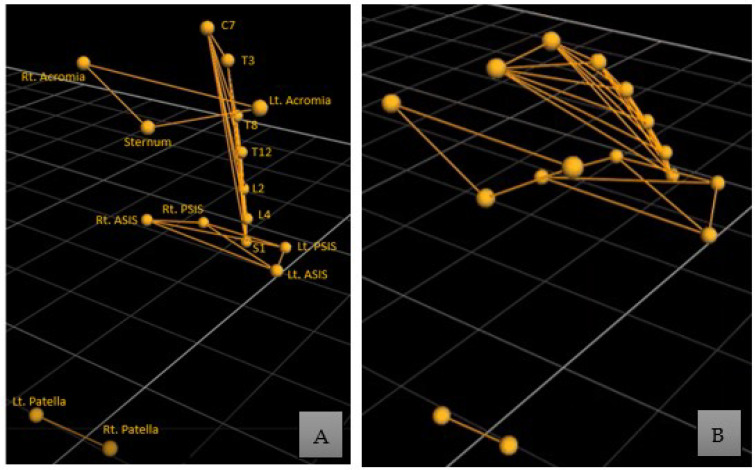
(**A**) Vicon markers positioned over the anatomical bony landmarks of a participant in a normal straight sitting position; (**B**) a flexion movement performed during the assessment.

The placement of the Vicon reflective markers and EMG electrodes was performed by an experienced physiotherapist. Specific bony landmarks were identified, and Vicon markers were placed as follows (Figure 2A): anteriorly, at the acromia, sternum, right and left anterior superior iliac spine (ASIS), and right and left patella; posteriorly, at the cervical spinous process (C7), thoracic spinous process (T3, T8, T12), lumbar spinous process (L2, L4), sacrum spinous process (S1), and right and left posterior superior iliac spine (PSIS) [12,48]. The EMG electrodes over the rectus abdominis muscle were positioned 5 cm below the xiphoid process, while for the external oblique they were placed 5 cm superior to the anterior superior iliac spine and 10 cm lateral to the umbilicus. Likewise, electrodes for the latissimus dorsi were placed 2 cm inferior and lateral to the scapula’s inferior angle, while those for the external oblique were placed 3 cm lateral to the L3 spinous process [49]. The tested movements included trunk flexion, trunk extension, bilateral trunk lateral flexion, and bilateral trunk rotation. Each movement was performed thrice, and the best one was selected for data analysis. All assessments were conducted without using TSCS. The placement of Vicon markers and a flexion movement performed in the assessment are shown in Figure 2.

### 2.6. Data Processing and Analysis

**Figure 3:**
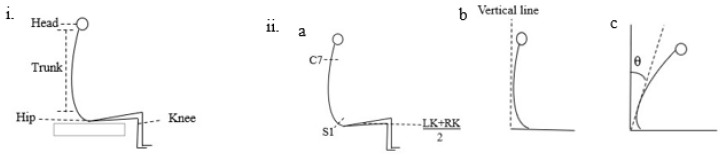
The principle of data analysis process of Vicon data for trunk flexion movement: (**i**) The erect sitting posture with knees in 90 degrees flexion and feet flat on the floor. For measuring flexion angle (**ii-a**) segments C7, S1, and the midline connecting the left knee (LK) and right knee (RK), (**ii-b**) an axis connecting the C7, S1 segments with a perpendicular line through the middle of the knees, (**ii-c**) flexion movement was performed, and angle measured in degrees (θ). The participant was allowed to take the required time to successfully and independently complete the movement. Similarly, measurements for other movements are presented in the Supplementary Materials (Supplementary Figure S1).

The functional outcome scores were graphically and statistically analyzed through GraphPad Prism version 9.0. Similarly, the kinesiologic and electrophysiologic data acquired from Vicon and EMG were extracted, processed, and analyzed using MATLAB (version 2016a, The MathWorks Inc., Natick, MA, USA). The sampling frequency was 2000 Hz and the filters applied were 10 Hz to 40 Hz during data collection. Different cutoff frequencies from 10 Hz to 50 Hz had been trialed, and 40 Hz was found to be the best based on visual inspection. The descriptive analysis (mean ± SD) was calculated for each phase. The trunk ROM angles were calculated by connecting selected markers at specific landmarks (Figure 3). A Friedman one-way repeated measures ANOVA with multiple comparisons was used to compare temporal differences in functional assessment (mFRT, TCT, and FIST) and ROM among the baseline, post-TSCS+TSR, post-TSR, and the final follow-up. The root mean square (RMS) value of EMG of each muscle was calculated in microvolts (µV), and the above-mentioned repeated measures of ANOVA was used to show significant improvements. In addition, Spearman correlation analysis was performed to determine the correlation between these assessment parameters (mFRT, TCT, FIST, EMG, and ROM). A *p*–value of 0.05 was set as the statistical level of significance.

## 3. Results

### 3.1. Combinational Treatment Initiated Functional Improvements in Reaching, Trunk Control, and Sitting Balance

**Figure 4:**
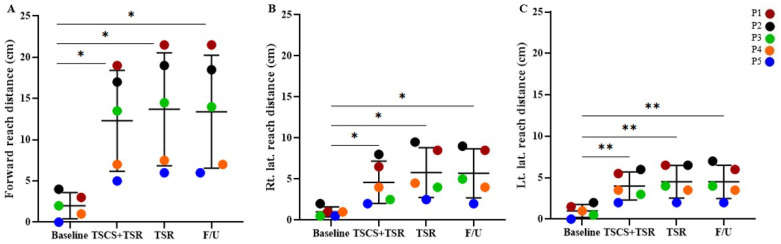
The increased functional scores of mFRT measured from the baseline. Statistical analysis was conducted between the baseline with TSCS+TSR, TSR, and follow-up. (**A**) Forward reach distance, (**B**) right lateral reach distance, and (**C**) left lateral reach distance, respectively, measured for each participant during the study. mFRT, Modified Functional Reach Test; TSCS, transcutaneous electrical spinal cord stimulation; TSR, task-specific rehabilitation; F/U, follow-up; * *p* < 0.05, ** *p* < 0.01, all in comparison to the baseline value.

As shown in Figure 4, the overall (mean ± SD) forward reach distance (FRD) was 2.0 ± 1.6 cm at the baseline, which increased by 10.3 ± 4.5 cm after TSCS+TSR (to 12.3 ± 6.1 cm), and further slightly raised by 1.4 ± 0.7 cm during TSR (to 13.7 ± 6.8 cm), which was maintained throughout the follow-up period in the absence of any intervention (to 13.4 ± 6.9 cm). Statistical analysis showed significant improvements between the baseline and TSCS+TSR (*p* = 0.025), TSR (*p* = 0.024), and follow-up (*p* = 0.026), respectively. For right lateral reach distance, the baseline mean ± SD was 0.9 ± 0.7 cm, which increased by 3.7 ± 1.8 cm after the introduction of TSCS+TSR (to 4.6 ± 2.6 cm), followed by a further increment of 1.2 ± 0.5 cm during the TSR training period (to 5.8 ± 3.0 cm), and then reduced to 5.7 ± 3.0 cm at the final follow-up. Significant improvements were found between the baseline and TSCS+TSR (*p* = 0.037), TSR (*p* = 0.029), and follow-up (*p* = 0.030), respectively. At the baseline, the left lateral reach distance was 1.0 ± 0.8 cm, which increased to 4.0 ± 1.7 cm after TSCS+TSR, and further increased to 4.5 ± 1.96 cm throughout TSR, and then remained constant during the follow-up period (4.5 ± 2.0 cm). Significant improvements were observed between the results of the baseline and TSCS+TSR (*p* = 0.014), TSR (*p* = 0.016), and follow-up (*p* = 0.017), respectively.

**Figure 5:**
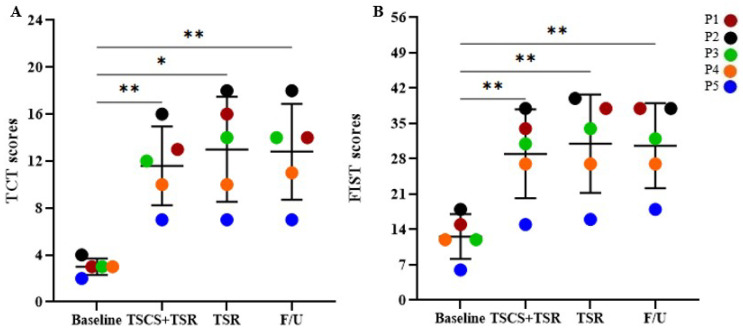
The increased functional scores of (**A**) TCT (0–24 points) and (**B**) FIST (0–56 points) measured from the baseline for each participant during the study. Statistical analysis was conducted between the baseline with TSCS+TSR, TSR, and follow-up. TCT, Trunk Control Test; FIST, Function in Sitting Test; TSCS, transcutaneous electrical spinal cord stimulation; TSR, task-specific rehabilitation; F/U, follow-up; * *p* < 0.05, ** *p* < 0.01, all in comparison to the baseline value.

As demonstrated in Figure 5A, the overall mean TCT score was 3.0 ± 0.7 at the baseline, which significantly increased to 11.6 ± 3.4 after TSCS+TSR administration, which increased further by 1.4 ± 1.1 during TSR to 13.0 ± 4.5, followed by a slight reduction to 12.8 ± 4.1 at the follow-up period. All these values of TCT were significantly greater than the baseline values (*p* < 0.01). Likewise, the overall mean FIST score (Figure 5B) was 12.6 ± 4.5 at the baseline, which underwent the greatest increase, by 29.0 ± 8.8, after TSCS+TSR, which further rose to 31.0 ± 9.7 during TSR, with a minor decrease of 0.4 ± 1.3 at the follow-up period (to 30.6 ± 8.4). All the FIST scores at the follow-ups were significantly larger than the baseline values (*p* < 0.01). However, there was no significant difference in the TCT or FIST scores between TSCS+TSR and TSR, between TSCS+TSR and follow-up, or between TSR and follow-up.

### 3.2. Increased Trunk Range of Motion after TSCS+TSR Treatment

**Figure 6:**
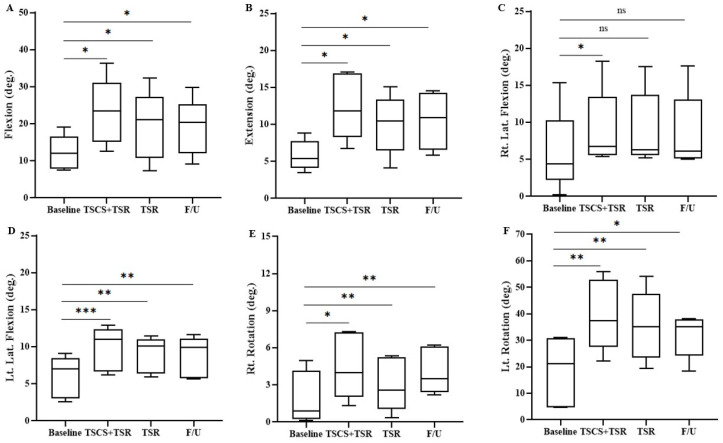
The trunk range of motion measured with Vicon for each participant during the study. Statistical analysis was conducted between the baseline with TSCS+TSR, TSR and follow-up. (**A**) Flexion, (**B**) extension, (**C**) right lateral flexion, (**D**) left lateral flexion, (**E**) right rotation, and (**F**) left rotation, respectively. TSCS, transcutaneous electrical spinal cord stimulation; TSR, task-specific rehabilitation; F/U, follow-up; * *p* < 0.05, ** *p* < 0.01, *** *p* < 0.001, non-significant (ns) *p* > 0.05, all in comparison to the baseline value.

Figure 6 shows that trunk flexion, extension, left lateral flexion, and bilateral rotation ROM at TSCS+TSR, TSR, and the final follow-up were significantly higher than the respective baseline values. However, the right trunk lateral flexion underwent significant improvement in ROM only after TSCS+TSR. Specifically, the overall trunk flexion ROM was 12.2° ± 4.7° at the baseline, which significantly increased to 23.1° ± 9.0° after TSCS+TSR, followed by a 3.7° ± 0.4° reduction in motion after TSR (to 19.4° ± 9.37°), which further decreased by 0.3° ± 1.7° to 19.1° ± 7.7° at the final follow-up. The trunk extension ROM at the baseline was 5.7° ± 2.0°, which was significantly increased to 12.4° ± 4.5° after TSCS+TSR, then back to 10.0° ± 4.0°, and then increased to 10.4° ± 3.9° at the follow-up. Likewise, the trunk right lateral flexion was 5.8° ± 5.6° at the baseline, which significantly increased to 9.1° ± 5.4° after TSCS+TSR, followed by a decrease to 8.4° ± 5.3° after TSR, and then to 8.7° ± 5.1° at the follow-up. At the baseline, the left lateral flexion ROM increased from 6.0° ± 2.8° to 9.8° ± 2.9° after TSCS+TSR, and then decreased to 8.8° ± 2.3° after TSR, and further dropped to 8.2° ± 2.6° at the follow-up. The right rotation increased from 1.7° ± 2.3° at the baseline to 4.5° ± 2.7° after the TSCS+TSR, then dropped to 3.0° ± 2.2° after TSR, followed by an increase to 4.1° ± 1.9° at the follow-up. The mean ± SD for left rotation was 18.4° ± 10.1° at the baseline, which increased by 21.2° ± 3.3° to 39.6° ± 13.4° after TSCS+TSR, followed by a decrease of 4.3° ± 0.2° to 35.3° ± 13.2° after TSR, and further reduction to 31.8° ± 8.2° at the follow-up. However, no significant difference was observed in any trunk ROMs between TSCS+TSR and TSR, between TSCS+TSR and follow-up, or between TSR and follow-up.

### 3.3. Elevated Electromyographic Response of the Trunk Muscles

The right latissimus dorsi (Rt LD) and left latissimus dorsi (Lt LD), as well as the right erector spinae (Rt ES) and left erector spinae (Lt ES) during extension, and the right external oblique (Rt EO) and left external oblique (Lt EO) during left rotation, exhibited the significantly highest EMG amplitude at TSCS+TSR and TSR than the respective baseline values in comparison to other trunk ROMs, i.e., flexion, bilateral lateral flexion, and right rotation. However, there was no significant difference observed in the EMG response at follow-up for Rt LD or Lt LD during extension, as well as for Rt EO and Lt EO for right rotation and left rotation, and Rt ES during left lateral flexion, respectively, compared with the baseline values (Table 2). The EMG responses of other trunk ROMs (excluding extension and left rotation) are presented in the Supplementary Materials (Supplementary Table S1). Specifically, the EMG response for extension was 2.20 ± 1.60 µV for Rt LD and 2.57 ± 1.81 µV for Lt LD at the baseline, which was significantly increased to 8.86 ± 6.04 µV (Rt LD) and 9.94 ± 6.70 µV (Lt LD), respectively, after TSCS+TSR, which further reduced to 5.01 ± 3.71 µV (Rt LD) and 5.70 ± 4.86 µV (Lt LD) after TSR. In addition, the EMG values remained almost unchanged after the follow-up for Rt LD (5.02 ± 3.03 µV) and Lt LD (6.07 ± 4.45 µV) in comparison with the baseline value, similar for the subsequent descriptions. Likewise, the EMG values during extension were 1.62 ± 0.95 µV (Rt ES) and 1.79 ± 1.25 µV (Lt ES) at the baseline, and increased to 6.93 ± 6.32 µV (Rt ES) and 7.53 ± 5.47 µV (Lt ES), respectively, after TSCS+TSR, followed by a further decrement to 4.11 ± 2.68 µV (Rt ES) and 4.57 ± 3.02 µV (Lt ES) after TSR. Additionally, the EMG amplitude remained almost unchanged after the follow-up for Rt ES (4.19 ± 2.58 µV) and Lt ES (4.69 ± 2.97 µV). For left rotation, the EMG response was 1.55 ± 0.93 µV for Rt EO and 2.07 ± 1.17 µV for Lt EO at the baseline, which significantly increased to 6.86 ± 3.94 µV (Rt EO) and 13.47 ± 7.49 µV (Lt EO) after TSCS+TSR, respectively, and further reduced to 6.06 ± 3.38 µV (Rt EO) and 12.06 ± 6.73 µV (Lt EO) after TSR, which remained almost consistent after the follow-up period for Rt EO (5.84 ± 3.40 µV) and Lt EO (11.19 ± 6.23 µV), respectively. Furthermore, no significant difference was observed in EMG amplitudes for any trunk ROMs between TSCS+TSR and TSR, between TSCS+TSR and follow-up, or between TSR and follow-up.

### 3.4. Improvements of Trunk Control and Function in Sitting after Combinational Treatment

**Figure 7:**
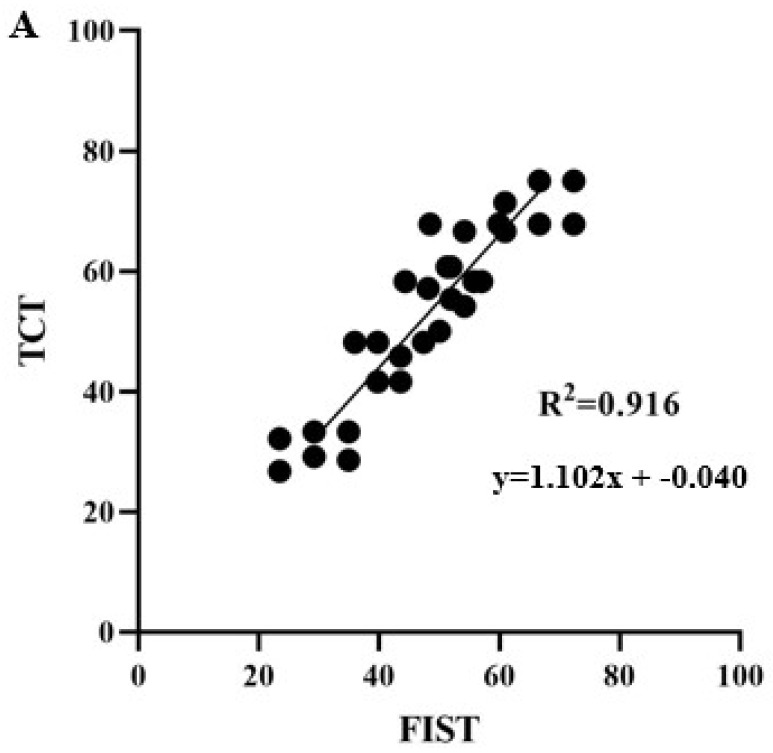
The correlation between the functional improvements regarding the Trunk Control Test (TCT) and Function in Sitting Test (FIST).

The functional improvements of trunk control and function in sitting were correlated with each other (Figure 7), demonstrating a strong positive correlation between the TCT and FIST after TSCS+TSR (R^2^ = 0.916, *p* = 0.001). Similarly, the TCT and mFRT (R^2^ = 0.774, *p* = 0.017), TCT and EMG (R^2^ = 0.743, *p* = 0.009), EMG and ROM (R^2^ = 0.626, *p* = 0.004), and FIST and EMG (R^2^ = 0.746, *p* = 0.0125), respectively, exhibited mild positive correlations after the TSCS+TSR. Conversely, FIST and mFRT (R^2^ = 0.305, *p* = 0.009), EMG and mFRT (R^2^ = 0.233, *p* = 0.047), and TSR and TSCS+TSR (R^2^ = 0.217, *p* = 0.094) displayed weak correlations following TSCS+TSR.

### 3.5. Treatment Effect on Sensorimotor Recovery

**Figure 8:**
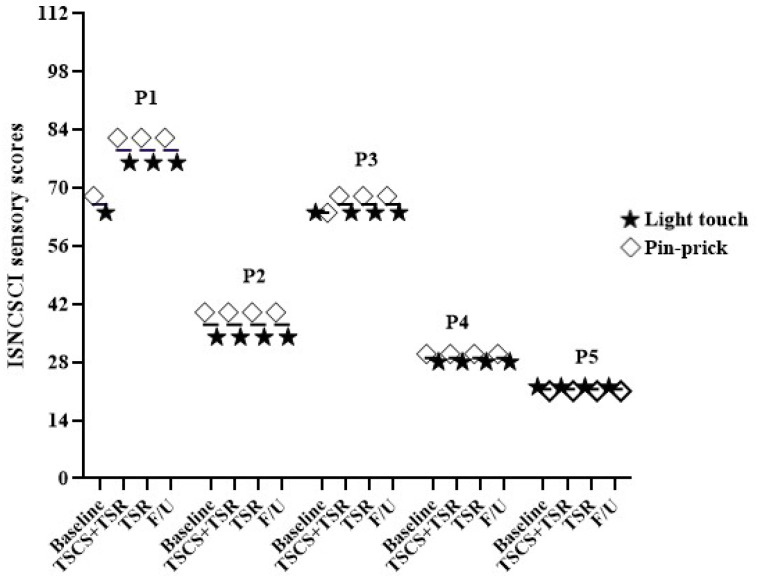
The International Standards for Neurological Classification of Spinal Cord Injury (ISNCSCI) scores with light touch and pinprick sensory sub-scores obtained in this study.

The ISNCSCI underwent some changes in the sensorimotor scores, but the neurological level of injury and AIS remained unchanged (Supplementary Table S2). Figure 8 shows that for ISNCSCI, a participant (P1) revealed an increase of 8 points and 18 points in response to light touch and pinprick sensation (68/64 to 76/82), respectively, while P3 showed a 4-point elevation in response to pinprick sensation (64/64 to 64/68) after TSCS+TSR. Moreover, throughout TSR and the follow-up period, the increased motor and sensory scores remained unchanged. However, over the entire study period, there was no change in ISNCSCI scores for P2, P4, or P5.

### 3.6. The Impact of Treatment Frequency on Functional Outcome

**Figure 9:**
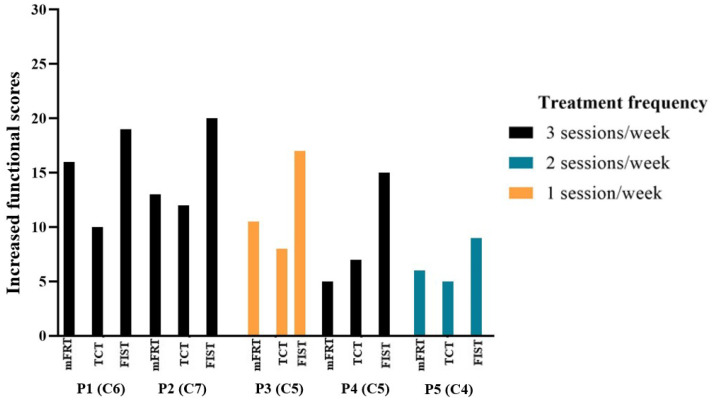
The increased functional scores based on treatment frequency obtained by the participants (cervical cord injury level: C4, C5, C6, C7) during the functional assessment of Modified Functional Reach Test (mFRT), Trunk Control Test (TCT), and Function in Sitting Test (FIST). A higher score indicates an improvement in function.

The frequency of treatment delivered to the study participants is illustrated in Figure 9, where P1, P2, and P4 received the intervention thrice per week with C6-, C7-, and C5-level cervical cord injuries, respectively. P3, who attended once a week, had a SCI at the C5 level. Furthermore, P5, who participated twice a week, had a C4 SCI. P1 and P2 improved the most in regard to mFRT (forward reach), TCT, and FIST. Additionally, P4, who attended the same treatment sessions as P1 and P2, presented with minimal improvement, relative to P1 and P2, in comparison to the increased functional scores. Interestingly, P3 scored better than P5 in terms of the above functional outcome measures. However, their injury levels differed from each other. Additionally, P3, who received fewer weekly sessions, improved more than P4, although they had the same injury level. Furthermore, P1 and P2 were able to independently perform transfers from their wheelchairs to their beds and vice versa, using a sliding board, under the supervision and assistance of caregivers. They also reported feeling more secure and having less fear of falling.

## 4. Discussion

The present investigation examined the effects of combining TSCS and TSR treatment on trunk control and sitting function in individuals with complete cervical SCI. The study demonstrates that the combined intervention (TSCS+TSR) could improve trunk stability along with static and dynamic sitting balance in people with complete tetraplegia. The results substantiate that all participants trunk control and sitting function progressively improved during TSCS+TSR, no matter whether they received one, two, or three treatment sessions per week with an injury at C4, C5, C6, or C7 level. In addition, functional improvements were maintained throughout the subsequent TSR and follow-up periods, demonstrating positive long-term treatment effects. Our findings highlight that trunk recovery is possible even in people with chronic complete cervical SCI, and the functional gains can be achieved using TSCS+TSR.

### 4.1. Functional Reaching Ability in Sitting

TSCS+TSR can enhance the forward reaching ability of people with SCI, which is essential in order for wheelchair users to accomplish everyday tasks [50]. The present study reported an increase in FRD (10.3 ± 4.5 cm) after TSCS+TSR, which enabled the participants to reach objects placed in front of them. Additionally, the lateral reach distances also increased to assist the maintenance of trunk balance. Prior research demonstrated that implanted electrical stimulation in SCI individuals with thoracic-level injuries exhibited improved FRD (5.5 ± 6.6 cm) in sitting [8]. Another study revealed that people with thoracic SCI receiving epidural stimulation displayed increased FRD, while lateral reach lengths remained unchanged [51]. The current results support these findings; additionally, the lateral reach distances also significantly increased. It has been previously revealed that those with higher FRD exhibited greater sitting control and postural stability [50]. Likewise, the current investigation indicates that participants with increased FRD demonstrated improved trunk and sitting function. This may assist clinicians to individualize exercise regimens to improve the wheelchair-based ADL of people with SCI based on their abilities [45]. Prior research showed that “off and on” epidural spinal cord stimulation settings affected the reach lengths, where the individuals reaching abilities returned to normal when the stimulator was switched off, but the FRD increased while the stimulator was on [51]. However, the present study shows that the increased reaching distances following TSCS+TSR were maintained even in the absence of further stimulation during the follow-up period.

### 4.2. Trunk Control and Seated Postural Stability

While falls have been reported in up to 75% of people with SCI, lack of trunk control is the predominant reason for falls in these individuals [52]. As trunk stability is a significant contributor to falls, rehabilitation programs should aim to increase sitting balance [52]. Interestingly, the present results indicate that TSCS+TSR could improve static trunk control in all participants, with the ability to sit independently with or without support of the upper limbs, while dynamic trunk control was restored in two participants. Therefore, improved trunk control could reduce the risk of falling from a wheelchair. Although a chest strap is beneficial for stabilizing the trunk of individuals with paraplegia in a wheelchair, it is only a temporary external method [53]. Given that TSCS+TSR could improve independent sitting and trunk function with increased FRD in people with tetraplegia, this method may complement or even replace traditional techniques such as chest straps, seating adjustments, and other manual support. The promising results suggest that this intervention may have the potential to help individuals with tetraplegia to turn in bed, which is critical in preventing the development of pressure injuries.

Sitting stability is essential for the functioning of those who cannot stand. The inability to complete transfer tasks may limit an individual’s independence in a wheelchair, thereby interfering with ADL [54]. It was thought that a decrease or increase in trunk strength might predict sitting balance in people with SCI, although this premise remains inconclusive [10]. Our outcomes show that individuals with a lower cervical SCI displayed greater recovery in seated function, trunk strength, and sitting stability than those with a higher cervical SCI. A recent study using TSCS also showed an immediate effect on the upright sitting ability of SCI individuals that improved trunk stability and maintained sitting balance [12]. However, participants in that study had less severe injuries, and the level of injury was lower than that of our participants. That said, three of our participants reported improvements in their ability to propel their wheelchairs. They switched from motorized wheelchairs to manual wheelchairs.

### 4.3. Trunk Muscle EMG Response and Range of Motion

TSCS can increase trunk muscle activity and active ROM. Individuals with SCI above the L1 level experience trunk instability due to impaired trunk musculature [55]. A recent study showed that TSCS significantly increased the EMG response of the ES, RA, and EO muscles, which promoted trunk stability and sitting balance in people with chronic SCI [12]. These findings concur with those of the current study. Although invasive functional electrical stimulation (FES) together with therapeutic exercise increased trunk muscle tone and improved dynamic sitting stability, the effects reverted without FES [56]. Conversely, our participants demonstrated increased EMG responses in ES, EO, LD, and trunk ROM (maximum for flexion and rotation) following 12 weeks of TSCS+TSR. These effects persisted even without further stimulation. Previous research showed that stimulating trunk muscles with TSCS for thoracic-level SCI resulted in an increased mean trunk extension by 9.2° ± 9.5° in sitting [8], whereas the current study yielded an even greater increased mean extension of 12.4° ± 4.5°. Collectively, these EMG changes may indicate the initial stage of neuromuscular recovery [36], which may result in further functional improvements with prolonged treatment. Interestingly, the right rotation (4.5° ± 2.7°) was significantly lower than the left rotation (39.6° ± 13.4°). A prior study reported that an individual who had right hand dominance before the SCI showed greater gains in relation to right hand function than the left side after bimanual task-oriented training, likely due to hand dominance [57]. As such, it was possible that hand dominance might contribute to the current findings. However, a recent study on sensorimotor and functional recovery between dominant and non-dominant upper extremities showed no significant differences in outcomes [58]. Therefore, further research is required to determine the veracity of this statement.

### 4.4. TSCS and TSR for Sensorimotor Improvement

In recent years, TSCS has become increasingly popular as a treatment option for people living with SCI. During locomotion training, TSCS has demonstrated exceptional gains in motor performance that were previously believed to be unattainable for individuals with chronic SCI [59]. Researchers have hypothesized that TSCS may have long-term benefits even in cases of complete SCI [12,42]. The TSCS treatment has shown continual improvements in locomotion [60], standing [44], upper extremity function [61], and sitting posture [12,42] in individuals with SCI. A few studies have also reported the immediate effects of TSCS on maintaining postural stability in people with SCI [12,42]. The present study adds to the findings of previous studies [12,42] by demonstrating that TSCS+TSR induced sustainable improvement in trunk function in people with complete tetraplegia. Our findings may assist clinicians and rehabilitation experts in the planning of trunk rehabilitation programs for individuals with complete tetraplegia to enhance patients’ trunk stability and sitting balance.

Furthermore, the effects of TSCS on the sensory functions of the study participants revealed that pinprick and light touch sensations were improved, particularly in P1, with no prognosis at the neurological level. This may be attributed to the fact that individuals with SCI often reach a plateau in their spontaneous physical recovery approximately 1.5 years after SCI [62]. While most of our participants were 2–19 years post-SCI, for P1, post-SCI injury duration was 1.5 years. This could be one of the potential causes of the sensory improvement in P1 only. Further studies are needed to confirm this hypothesis. A prior study reported similar outcomes, where no change in ISNCSCI scores was observed in individuals with complete SCI (AIS A) [44]. Some studies showed improvements in neurological levels. However, the recovery was demonstrated in people with incomplete SCI [61,63]. Simultaneously, motor function improvements have been observed in individuals with complete SCI, but evidence on clinical prognosis is lacking [61].

The present study also shows that people with complete tetraplegia are unlikely to recover by TSR alone. This statement is supported by a review study evaluating the effectiveness of TSR for improving independent sitting and standing function in people with chronic SCI, which highlighted the minimal effect of TSR on functional recovery [29]. Indeed, little benefit to motor improvement has been reported in people with incomplete SCI [30], and another review paper presented little evidence on the benefit of conventional therapeutic exercise for increasing motor strength after SCI [64]. The current study findings support the above statements, where no significant improvements were observed when the TSR treatment was administered alone.

Research suggests that the combination of neuromodulation with a specific rehabilitation approach may recover adequate function after SCI [65]. The current findings support this notion, where significant improvements were seen after TSCS+TSR. Interestingly, the findings indicate that TSCS with TSR increased trunk stability and improved sitting balance, which assisted the study participants to advance the functional tasks (such as being able to perform rolling, accomplish transfer tasks, achieve progression in spinal mobility functions, and maintain erect sitting posture) independently or with minimal assistance. The impact of treatment frequency on functional outcome shows that improvement varied between individuals. These differences in existing functional outcome may be related to SCI level. Participants with lower tetraplegia had significantly more improvement than those with higher tetraplegia, despite attending similar treatment sessions. Given that a participant (P3) with lower cervical SCI, who attended fewer treatment sessions than another participant (P1) with higher cervical SCI, had better improvements, it is possible that improvement depends on the SCI level of neurological damage rather than the frequency of treatment. Future research should evaluate whether continuous intervention sessions may yield further recovery.

### 4.5. Relationship between Functional Improvement of mFRT, TCT, and FIST

The TCT and FIST were found to have a stronger correlation than other assessment parameters. Both TCT and FIST assessments have several trunk-based static and dynamic balancing assessments. These activities provide a more stable sitting position [46,66]. Therefore, when the trunk control is increased, the sitting function will also improve. This may explain their strong correlation in the current study. The mFRT did not show a strong correlation with other outcome measures. Daily activities or functional movements require a wide range of motions. Forward or lateral body movements alone may not accurately reflect the functional ability of an individual [16]. A prior study also found no correlation between mFRT and the mobility assessment of spinal cord independence measure III in people with chronic SCI [16]. Similarly, the lack of correlation between ROM and other functional assessment parameters in our study may be because SCI individual’s ability to flex or extend their trunks had no effect on sitting stability [10]. Specifically, prolonged sitting in a wheel chair may lead to the development of a C-shaped spinal sagittal profile in people with SCI due to hyperkyphosis, hypolordosis, and a posterior pelvic tilt, which could affect sitting stability [9,67]. In fact, trunk muscle strength and endurance [68], pelvic alignment [69], and forward reaching ability together are the determinants of seated performance [70]. Therefore, improved ROM alone may not result in the corresponding improvement in sitting ability.

### 4.6. Study Limitations and Indications for Future Research

The research described herein has several limitations. First, the participants had varying levels of tetraplegia, which affected their ability to perform motor tasks to different extents. Although motor improvements were observed in all of the study participants regardless of the number of treatment sessions, greater functional gain was observed in those with lower levels of tetraplegia. Second, the participants were recruited via convenience sampling, which might lead to selection bias. Third, because one participant with C4 SCI (AIS A) had a sudden increase in blood pressure, the training was hampered by the need for frequent pauses to avoid autonomic dysreflexia. Fourth, one participant developed skin rashes after stimulation, which required a week to recover, necessitating the suspension of training for a week. To successfully complete all sessions, the treatment time was prolonged for that participant. Fifth, because the data collection was performed by the same investigator, there was a possibility of some bias. It was difficult to blind the participants because they underwent the treatment. Given the difficulty of double blinding, we adopted single blinding, where the rater was blinded in order to reduce the potential bias.

Despite our promising results, the mechanisms underlying the combined treatment effects remain unknown. One possible mechanism could be that supraspinal adaptations significantly improve balance performance following externally challenged balance training which is facilitated by a feed-forward mechanism [11]. Since the posterior root reflexes were not studied, the investigators did not know which structures were activated by the stimulation and parameters used [43]. Future studies should investigate the mechanisms and posterior root reflexes. Furthermore, future research should validate the current findings in a larger sample from a wider geographic region. Randomized controlled trial studies are warranted to compare the effects of TSCS+TSR and TSR alone under different treatment regimens with adjustment for various confounders (e.g., body mass index, duration of injury, residual upper limb function, and grasp function) in order to inform clinical practice. Future work should also evaluate the effects of TSCS+TSR on the quality of life of people with chronic complete cervical SCI.

## 5. Conclusions

The current study demonstrates that 12 weeks of TSCS+TSR treatment significantly improved functional reach distance and trunk muscle activation in multiple directions among individuals with chronic complete cervical SCI. These improvements persisted even after TSCS had been stopped for 18 weeks. The resulting increase in sitting balance may reduce the risk of falling from a wheelchair and improve functional independence when carrying out the purposeful tasks in sitting. The findings may be considered preliminary, and future studies should determine the optimal treatment regimen and duration to attain the maximum long-term treatment effects.

## Figures and Tables

**Table 1:** The demographic and clinical characteristics of the participants.

Participants	Age	Gender	Time since Injury (Years)	Type of Injury	NLI	AIS Category
P1	57	F	1.5	Traumatic	C6	A
P2	55	F	19	Traumatic	C7	A
P3	26	F	12	Traumatic	C5	A
P4	40	M	12	Traumatic	C5	A
P5	32	M	2	Traumatic	C4	A

NLI, neurological level of injury; AIS, American Spinal Injury Association Impairment Scale.

**Table 2:** The responses recorded from trunk muscles measured through EMG.

**(A)**								
**Study Timeline**	**Extension**							
	**Rt. ES**		**Lt. ES**		**Rt. LD**		**Lt. LD**	
	**Mean ± SD**	***p*-Value**	**Mean ± SD**	***p*-Value**	**Mean ± SD**	***p*-Value**	**Mean ± SD**	***p*-Value**
Baseline	1.62 ± 0.95	-	1.79 ± 1.25	-	2.20 ± 1.60	-	2.57 ± 1.81	-
TSCS+TSR	6.93 ± 6.32	*	7.53 ± 5.47	*	8.86 ± 6.04	***	9.94 ± 6.70	*
TSR	4.11 ± 2.68	***	4.57 ± 3.02	***	5.01 ± 3.71	***	5.70 ± 4.86	*
Follow-up	4.19 ± 2.58	***	4.69 ± 2.97	***	5.02 ± 3.03	ns	6.07 ± 4.45	***
**(B)**								
**Study timeline**		**Left rotation**						
		**Rt. EO**			**Lt. EO**			
		**Mean ± SD**		***p*** **-Value**	**Mean ± SD**		***p*** **-Value**	
Baseline		1.55 ± 0.93		-	2.07 ± 1.17		-	
TSCS+TSR		6.86 ± 3.94		*	13.47 ± 7.49		*	
TSR		6.06 ± 3.38		*	12.06 ± 6.73		*	
Follow-up		5.84 ± 3.40		*	11.19 ± 6.23		*	

LD, latissimus dorsi; ES, erector spinae; EO, external oblique; Rt, right; Lt, left; Lat, lateral; SD, standard deviation; TSCS, transcutaneous electrical spinal cord stimulation; TSR, task-specific rehabilitation; * *p* < 0.05, *** *p* < 0.001, non-significant (ns) *p* > 0.05, all in comparison with the baseline value.

## Data Availability

The data generated from this work can be obtained from the corresponding author upon reasonable request.

## Supplementary Materials

The following supporting information can be downloaded at: https://www.mdpi.com/article/10.3390/biomedicines11010034/s1, Figure S1: The detailed analysis process of Vicon involves connecting selected markers at specific anatomical landmarks; Table S1: The responses recorded from trunk muscles measured through EMG; Table S2: The ISNCSCI classification of the participants.
