# Supplementary material for: Combined Transcutaneous Electrical Spinal Cord Stimulation and Task-Specific Rehabilitation Improves Trunk and Sitting Functions in People with Chronic Tetraplegia
Source: Biomedicines. 2022 Dec 23;11(1):34. doi: 10.3390/biomedicines11010034 (PMC9855778; doi:10.3390/biomedicines11010034)
Supplement: Supplementary file 1 [file biomedicines-11-00034-s001.zip › biomedicines-2110180-supplementary.pdf]

Supplementary Materials:

Table S1. The responses recorded from trunk muscles measured through EMG.

A

Study timeline	Flexion							
	Rt. RA		Lt. RA		Rt. LD		Lt. LD	
	Mean ± SD	*p*-value	Mean ± SD	*p*-value	Mean ± SD	*p*-value	Mean ± SD	*p*-value
Baseline	0.66 ± 0.27	-	1.23 ± 0.56	-	2.65 ± 1.82	-	2.90 ± 1.85	-
TSCS+TSR	1.30 ± 0.60	**	2.27 ± 0.16	*	6.54 ± 4.26	***	7.14 ± 4.49	***
TSR	1.39 ± 0.26	***	1.57 ± 0.32	***	4.82 ± 2.94	***	5.11 ± 2.76	***
Follow-up	1.39 ± 0.24	ns	1.50 ± 0.30	***	4.97 ± 2.90	***	5.15 ± 2.55	***

B

Study timeline	Right lateral flexion							
	Rt. EO		Lt. EO		Rt. ES		Lt. ES	
	Mean ± SD	*p*-value	Mean ± SD	*p*-value	Mean ± SD	*p*-value	Mean ± SD	*p*-value
Baseline	1.96 ± 1.0	-	3.22 ± 2.09	-	2.0 ± 1.98	-	1.53 ± 1.64	-
TSCS+TSR	3.24 ± 2.54	***	9.15 ± 6.71	***	6.89 ± 4.57	*	5.19 ± 2.76	*
TSR	3.11 ± 1.95	***	7.76 ± 5.37	***	6.33 ± 4.63	ns	5.02 ± 3.07	*
Follow-up	3.0 ± 1.84	***	7.77 ± 5.11	***	6.43 ± 4.70	ns	5.12 ± 2.86	*

C

Study timeline	Left lateral flexion							
	Rt. EO		Lt. EO		Rt. ES		Lt. ES	
	Mean ± SD	*p*-value	Mean ± SD	*p*-value	Mean ± SD	*p*-value	Mean ± SD	*p*-value
Baseline	1.10 ± 0.79	-	2.81 ± 2.60	-	1.40 ± 1.17	-	2.02 ± 1.70	-
TSCS+TSR	2.06 ± 1.55	***	7.80 ± 5.64	*	5.32 ± 3.34	*	6.46 ± 4.38	***
TSR	2.36 ± 1.39	***	7.11 ± 4.65	*	5.69 ± 4.65	ns	5.92 ± 4.68	***
Follow-up	2.34 ± 1.30	***	6.94 ± 4.61	ns	5.53 ± 4.22	ns	6.01 ± 4.62	***

D

Study timeline	Right rotation			
	Rt. EO		Lt. EO	
	Mean ± SD	*p*-value	Mean ± SD	*p*-value
Baseline	1.75 ± 1.31	-	2.42 ± 1.69	-
TSCS+TSR	6.15 ± 4.83	*	9.97 ± 6.08	*
TSR	5.62 ± 4.34	ns	9.41 ± 4.96	*
Follow-up	5.51 ± 4.15	ns	9.08 ± 4.82	*

(LD: latissimus dorsi, ES: erector spinae, EO: external oblique, Rt: right, Lt: left, Lat: lateral, * $p < 0.05$, ** $p < 0.01$, *** $p < 0.001$, non-significant (ns) $p > 0.05$, all in comparison with the baseline value).

Table S2. The ISNCSCI classification of the participants.

Participants	P1	P2	P3	P4	P5
			**NLI, AIS**		
			**Motor Level Right/Left**		
Baseline	C6/A	C7/A	C5/A	C4/A	C5/A
	C6/C6	C8/C7	C6/C5	C5/C5	C6/C5
TSCS+TSR	C6/A	C7/A	C5/A	C4/A	C5/A
	C6/C6	C8/C7	C6/C5	C5/C5	C6/C5
TSR	C6/A	C7/A	C5/A	C4/A	C5/A
	C6/C6	C8/C7	C6/C5	C5/C5	C6/C5
Follow-up	C6/A	C7/A	C5/A	C4/A	C5/A
	C6/C6	C8/C7	C6/C5	C5/C5	C6/C5
			**Light touch/Pin prick**		
Baseline	68/64	40/34	64/64	28/30	22/21
TSCS+TSR	**76**/**82**	40/34	64/**68**	28/30	22/21
TSR	**76**/**82**	40/34	64/**68**	28/30	22/21
Follow up	**76**/**82**	40/34	64/**68**	28/30	22/21

NLI: Neurological Level of Injury; AIS: American Spinal Injury Association Impairment Scale; light touch and pin prick sensation (each 0 – 112 points).

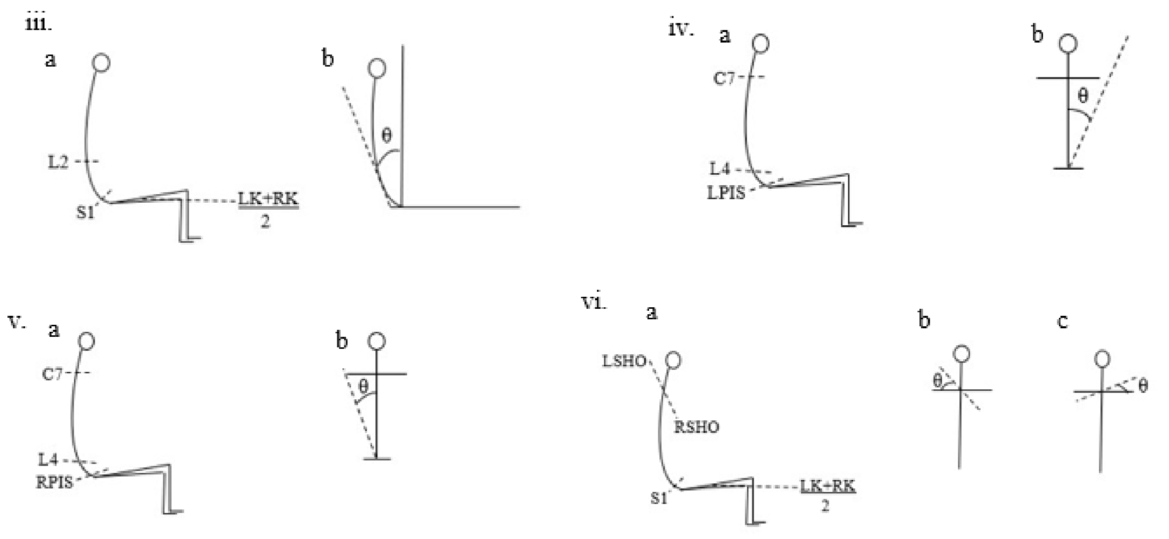

Figure S1. The detailed analysis process of Vicon involves connecting selected markers at specific anatomical landmarks. For extension movement (iii-a) segments L2, S1 and mid-line joining both knees (LK and RK), (iii-b) an axis connecting the L2, S1 segments with the perpendicular line through the middle of the knees, demonstrated the angle measured in degrees that was performed during extension of the trunk, (iv-a) segments C7, L4 and left posterior iliac spine (LPIS) were used to measure left lateral flexion LLF, (iv-b) an axis joining the segments where LLF motion was performed and revealed through measured angle (θ), (v-a) segments C7, L4 and right posterior iliac spine (RPIS) were used to assess right lateral flexion (RLF), (v-b) an axis connecting the segments where RLF motion was performed and revealed through measured angle (θ), (vi-a) an axis joining the left shoulder (LSHO) and right shoulder (RSHO) with S1 segment meeting to a straight line drawn through the mid-point of bilateral knees, (vi-b) left rotation movement was performed and shown through measured angle (θ), (vi-c) right rotation motion was performed and revealed via measured angle (θ).
